# Effect of Tachinid Parasitoid *Exorista japonica* on the Larval Development and Pupation of the Host Silkworm *Bombyx mori*

**DOI:** 10.3389/fphys.2022.824203

**Published:** 2022-02-16

**Authors:** Min-Li Dai, Wen-Tao Ye, Xue-Jian Jiang, Piao Feng, Qing-Yu Zhu, Hai-Na Sun, Fan-Chi Li, Jing Wei, Bing Li

**Affiliations:** ^1^School of Basic Medicine and Biological Sciences, Soochow University, Suzhou, China; ^2^Guangxi Academy of Forestry Sciences, Naning, China; ^3^Sericulture Institute of Soochow University, Suzhou, China

**Keywords:** tachinid parasitoid, *Bombyx mori*, pupation metamorphosis, 20E signaling, *Ftz-f1*

## Abstract

The Tachinidae are natural enemies of many lepidopteran and coleopteran pests of crops, forests, and fruits. However, host-tachinid parasitoid interactions have been largely unexplored. In this study, we investigated the effects of tachinids on host biological traits, using *Exorista japonica*, a generalist parasitoid, and the silkworm *Bombyx mori*, its lepidopteran host, as models. We observed that *E. japonica* parasitoidism did not affect silkworm larval body weight gain and cocooning rate, whereas they caused shortened duration of molting from the final instar to the pupal stage, abnormal molting from larval to pupal stages, and a subsequent decrease in host emergence rate. Moreover, a decrease in juvenile hormone (JH) titer and an increase in 20-hydroxyecdysone (20E) titer in the hemolymph of parasitized silkworms occurred. The transcription of JH and 20E responsive genes was downregulated in mature parasitized hosts, but upregulated in parasitized prepupae while Fushi tarazu factor 1 (*Ftz-f1*), a nuclear receptor essential in larval ecdysis, showed dramatically reduced expression in parasitized hosts at both the mature and prepupal stages. Moreover, the transcriptional levels of *BmFtz-f1* and its downstream target genes encoding cuticle proteins were downregulated in epidermis of parasitized hosts. Meanwhile, the content of trehalose was decreased in the hemolymph, while chitin content in the epidermis was increased in parasitized silkworm prepupae. These data reveal that the host may fine-tune JH and 20E synthesis to shorten developmental duration to combat established *E. japonica* infestation, while *E. japonica* silences *BmFtz-f1* transcription to inhibit host pupation. This discovery highlights the novel target mechanism of tachinid parasitoids and provides new clues to host/tachinid parasitoid relationships.

## Introduction

The Diptera Tachinidae are second only to the parasitic Hymenoptera (e.g., Ichneumonoidea and Chalcidoidea) in diversity with around 8,500 species described worldwide and ecological importance as parasitoids and are among the most species rich of Diptera families (O’Hara, 2013; Stireman, 2016). They attack hosts across at least 15 orders of Arthropods (e.g., Lepidoptera, Heteroptera, Coleoptera, Hymenoptera, and Orthoptera) and widely contribute to pollination success (Stireman et al., 2019; Raguso, 2020). Approximately, 70% of the tachinid species parasitize larval Lepidoptera and some of them have been employed in biological control programs of crop and forest pests (Elkinton and Boettner, 2012; Jiang et al., 2016). Despite their importance in ecological impact and pest management, tachinids as a group are far less studied than parasitic hymenopterans, probably as a consequence of their large size relative to wasp parasitoids, which is unfavorable for mass production (Dindo and Grenier, 2014). Although the tachinid biology and their application in controlling pests in managed agricultural and forest systems have been demonstrated, many gaps still exist in our understanding in tachinid parasitoid-host interactions from the perspective of ecology, physiology, and genomics (Dindo, 2011; Stireman, 2016). Advancing our understanding of their interactions will help to develop strategies that enhance the efficacy of tachinid parasitoids in biological pest control.

All the tachinids have three larval instars and may be solitary or gregarious and adopt diverse oviposition strategies, depending on the species (Dindo, 2011). Generally, most tachinid females oviposit their eggs directly on or indirectly near the host larvae and successful parasitoidism occurs when the parasitoid completes its development up to the adult stage. Subsequently, tachinid larvae develop in their host and pupate inside or outside host remains (Stireman, 2016). The early-instar tachinid larvae appear to avoid host immune defenses by forming respiratory funnels through which they maintain contact with outside air or enter in a host muscle or even a ganglion, unlike hymenopterans that are encapsulated by host immune cells, the lamellocytes, which lead to the death of developing wasps and no successful parasitoidism would occur (Anderl et al., 2016; Yamashita et al., 2019). Obviously, once the host is parasitized, the larva has to exploit host resource. In fact, competition for nutritional resources between the host and the larval parasitoid is complex. The larva must overcome host immune response and regulate host environments to meet its developmental needs. Definitely, in most cases, the parasitized hosts have no reproduction potential and do not survive the interaction (Dindo, 2011). In unsuitable hosts, even if host acceptance was successful, tachinids died whether for encapsulation or for unsuitability of the host resources (Rath and Sinha, 2005). During tachinid larval development, the host often exhibits altered development, reproduction, and behavior (Simões et al., 2004; Laws and Joern, 2012). For example, the larvae of *Helicoverpa armigera* parasitized by the tachinid *Chaetophthalmus dorsalis* entered the prepupal stage earlier than nonparasitized larvae and underwent normal behaviors associated with pupation and cocooning (Walker, 2011). However, the mechanism underlying these changes has rarely been investigated.

The parasitoid-induced developmental alterations are almost always associated with changes in host endocrine physiology. It appears that many koinobiont hymenopterans directly regulate the endocrine system of their hosts, either by inducing delayed or precocious initiation of metamorphosis, but later blocking completion of metamorphosis (Pennacchio et al., 2014; Riddiford and Webb, 2014). Concurrently, the two major insect hormones, juvenile hormone (JH) and 20-hydroxyecdysone (20E), are orchestrated to influence the development of insects (Kamruzzaman et al., 2020). In general, changes in the JH titer are due to alterations in JH synthesis by the corpora allata of the host and in the activity of host metabolic enzymes such as JH esterase (JHE). Alterations in host ecdysteroid titers usually trigger a cascade of 20E-responsive genes [i.e., transcription factor genes *BrC*, *E74*, *E75*, *Hr3*, *E93*, and Fushi-tarazu factor 1 (*Ftz-f1*)] after binding with the ecdysone receptor/ultraspiracle (EcR/USP) receptor complex, causing disturbance in development and metamorphosis (Lin et al., 2017; Elgendy et al., 2021). For example, parasitoids in the genus *Chelonus* induce precocious onset of metamorphosis followed by developmental arrest in prepupa of the lepidopteran hosts through regulating host JH and ecdysteroid production (Pfister-Wilhelm and Lanzrein, 1996; Jones et al., 2010). Teratocyte-secreting proteins or microRNAs of parasitic wasps could arrest host growth by altering host endocrine signals such as inhibiting synthesis of JHE and EcR (Ali et al., 2013; Wang et al., 2018). Thus, parasitiods either target host synthesis of JH and 20E or the downstream transcription activity of the two hormones to benefit their own development. Given that tachinids and their hosts have a long-term coexistence and coevolution history in nature, as a consequence, we anticipate that tachinid flies whose development are dependent of host physiology modulate host endocrine physiology to satisfy their developmental demands.

The tachinid fly *Exorista japonica* (Townsend) is widely distributed in China, India, Nepal, Vietnam, Japan, and Korea. It is a polyphagous parasitoid that attacks larvae of around 18 lepidopteran families, particularly noctuid larvae such as *Mythimna separata*, *Spodoptera litura*, and *Hyphantria cunea* (Li et al., 2012; Dindo and Nakamura, 2018; Seo et al., 2019). Therefore, it has great potential to control various pests of crops, forests, and fruit trees and, thus, of economic importance. The female fly lays eggs directly on the host cuticle. The first instar emerges and penetrates the host integument after incubation for few days (Dindo and Nakamura, 2018). This tachinid parasitoid takes 18–38 days to complete one generation when rearing on the common armyworm *Pseudaletia separata* or *H. cunea* depending on environmental conditions (Li et al., 2012; Dindo and Nakamura, 2018). *Bombyx mori*, the domesticated silkworm with low breeding cost, short generation time, and clear genetic background, is a major insect model for research (Meng et al., 2017). *E. japonica* is one of the natural enemies of *B. mori* and causes damage to sericulture industry^1^ (Gahukar, 2014). However, the pathogenic mechanism of *E. japonica* infection on the development of *B. mori* has not been clarified. In this study, we measured the performance of *E*. *japonica* on *B. mori*, investigated the influence of its parasitoidism on host development, and provided the possible underlying molecular mechanisms. This study provides insights into host relationships of Tachinidae, utilization of these beneficial insects in applied biological control, and management of parasitic tachinid flies in sericulture.

## Materials and Methods

### Insect Rearing

The silkworm *B. mori* strain Jingsong was stocked in our laboratory. Silkworm larvae were fed with fresh mulberry leaves (mulberry variety, Yu711) and reared in a transparent plastic box (30 cm in length, 10 cm in height, and 20 cm in width), which can accommodate 50 individuals in insect room at 25 ± 1°C under a photoperiod of 12:12 h (light/dark) and relative humidity of 75 ± 5% (Fang et al., 2021). The tachinid fly *E. japonica* was collected on its host, the *M. separata* in the field in Nanning, Guangxi, China. The rearing of *E. japonica* was followed by the method developed for the tachinid parasitoid *Exorista larvarum* (Diptera: Tachinidae) (Dindo et al., 2019). Briefly, newly emerged adults were fed with 20% honey solution; after mating, each of the fly cages was loaded with 100 silkworm larvae on day 1 at the last (fifth) instar for receiving fly eggs. The flies complete their larval stages within the silkworm body.

### Observation of the Life Parameters of *Exorista japonica* on Silkworms

Around 120 silkworms (sex ratio F/M = 1:1) on day 1 in the fifth instar stage were placed in the adult fly cages to receive fly eggs for 2 h. Due to 64% parasitoidism success in silkworms, silkworms received 2–3 fly eggs were selected and reared with mulberry leaves for further observation (Zhao et al., 2020). The newly hatched tachinid larvae penetrated the host body and each formed a visible black-marked respiratory funnel on the silkworm integument (Dindo et al., 2019). The number of silkworms with one and two funnels, the emerged tachinid puparia, and adults were recorded. The periods of tachinid egg-larva, egg-pupa, and pupa-adult were recorded.

### Observation of Silkworm Performance and Tissue Collection

A total of 100 silkworms (sex ratio F/M = 1:1) on day 1 in the fifth instar stage were placed in fly cages for receiving eggs for 2 h. To observe silkworm performance following tachinid parasitoidism, silkworms received 2–3 fly eggs were maintained for further observation and 50 silkworms not exposed to flies were observed as controls. For the treated larvae, body masses were measured at 4 and 6 days (day 5 and 7, fifth instar) after egg laying. For the control larvae, body masses were recorded at the same time intervals with the parasitized ones. Meanwhile, for both of the control and parasitized hosts, the duration of molting from the fifth instar to pupal stage, cocooning rate, pupation rate, and emergence rate were recorded. The number of parasitized silkworms that harbored alive tachinid larvae was also recorded. Additionally, the fat body, hemolymph, and epidermis samples from each silkworm individual at 4 and 8 days after parasitization (DAP) in the control and parasitized groups were collected (Supplementary Figure 1). Six silkworm tissues (sex ratio F/M = 1:1) were pooled as one replicate and three tissue replicates for each trial were prepared and stored in −80°C for further gene expression and metabolite analysis.

### Reverse Transcription-Quantitative PCR Analyses

For temporal expression analysis of JH- and 20E-responsive genes, including *BmJHE*, *BmEcR-A*, *BmEcR-B*, *BmUSP*, *BmE74*, *BmE75*, *BmHr3*, *BmBrC*, *BmE93*, and *BmFtz-f1*, and genes related to chitin synthesis and cuticle maintenance, including *BmTPS* (trehalose-6-phosphate synthase), *BmChsA* (chitin synthase A), *BmorCPG1*, *BmorCPR99*, *BmorCPR45*, *BmorCPR55*, *BmorCPR93*, *BmorCPG12*, and *BmorCPH2*, total RNA was extracted from the fat body, a cellular structure allowing efficient metabolic communication, and the epidermis of the control silkworm larvae and parasitized silkworms at 4 and 8 DAP using TRIzol reagent (catalog no. 15596018; Ambion Incorporation, Austin, TX, United States) and complementary DNAs (cDNAs) were generated with oligo (dT) and random primers using the PrimeScript RT Reagent Kit with genomic DNA (gDNA) Eraser (catalog no. RR047A; Takara Biotechnology Incorporation, Kusatsu, Shiga, Japan). Reverse transcription-quantitative PCR (RT-qPCR) was performed using the ViiA 7 Real-time PCR System (Applied Biosystems, Foster City, CA, United States). Total RNA was reverse-transcribed in a 20-μl reaction with SYBR Premix Ex Taq*™* II (catalog no. RR820A; Takara, Biotechnology Incorporation, Kusatsu, Shiga, Japan). The RT-qPCR protocol was 95°C for 1 min, followed by 40 cycles of 95°C for 5 s, 60°C for 10 s, and 72°C for 10 s. The results (threshold cycle values) of the RT-qPCR assays were normalized to the expression level of *B. mori* Actin3 gene (Anitha et al., 2014). The relative expression levels of genes were calculated using the 2^–ΔCt^ method. The primers of the tested genes are shown in Supplementary Table 1 (Anitha et al., 2014; Shahin et al., 2016; Chen et al., 2020).

### Analysis of Juvenile Hormone and Ecdysone Content

Hemolymph was collected from each individual of control and parasitized silkworms at 4 and 8 DAP by cutting an abdominal leg on ice. For each silkworm, a volume of 200 μl of hemolymph was collected. Each tested hemolymph sample (1,200 μl in total) was pooled by hemolymph from six individuals and then was centrifuged at 12,000 rpm for 10 min at 4°C. A volume of 10 μl of the supernatants was dissolved in ELISA buffer. The levels of JH III and 20E in the hemolymph were determined using the commercial JH III Enzyme Immunoassay Kit (Lvye Biotechnology, Suzhou, China) and the commercial 20E Enzyme Immunoassay Kit (Cayman, Ann Arbor, United States) according to the instructions of the manufacturer. The absorbance at 450 nm was determined and the standard curve was plotted using their standards (Sigma-Aldrich, St Louis, MO, United States). This experiment was replicated three times.

### Analysis of Trehalose Content and Trehalase Activity

The same hemolymph samples used for JH and 20E content analysis were used for measurement of trehalose levels. A volume of 10 μl of each hemolymph samples was used for the assay using the commercial insect Trehalose ELISA Kit (catalog no. MM3440901, Lvye Biotechnology, Suzhou, China). The absorbance at 450 nm was checked according to the instructions of the manufacturer. The content was calculated based on a standard curve and expressed as a ratio of trehalose production to fresh hemolymph (ng/ml). This experiment was performed in triplicate.

Trehalase activity was investigated with the commercial Trehalase Assay Kit (catalog no. BC2515, Solarbio, Beijing, China). One milliliter hemolymph was ultrasounded in a 50-ml plastic centrifuge tube with an ultrasonic cell disintegrator of 600 W ultrasonic power, 5 s interval time, 30 s ultrasonic time, and 20 min total working time. The samples were kept in an ice bath during the ultrasonic process to prevent overheating. Then, the ultrasonicated hemolymph was centrifuged at 12,000 rpm for 15 min at 4°C and the supernatant and precipitate were regarded as the fractions containing soluble (trehalase 1, BmTreh1) and membrane-bound trehalase (trehalase 2, BmTreh2), respectively. A volume of 100 μl of the supernatant (BmTreh1) and the precipitate (BmTreh2) were suspended with 100 μl phosphate-buffered saline (PBS) and the activity was measured according to the instructions of the manufacturer (Kamei et al., 2011). Trehalase activity was expressed as a ratio of trehalase activity to fresh hemolymph (U/ml). The enzymatic activity was determined in triplicate.

### Analysis of Chitin Content

The method for chitin measurement was performed as previously described and modified (Xu et al., 2020). Briefly, epidermis samples from silkworm prepupae were collected and homogenized in 400 μl 3% sodium dodecyl sulfate (SDS), incubated at 100°C for 15 min and centrifuged, then washed the precipitate with double distilled H_2_O (ddH_2_O) and resuspended in 400 μl 14 mol/l KOH, incubated at 130°C for 1 h. Then, the sample was mixed with 800 μl ice-cold 75% ethanol, incubated on ice for 15 min, and then mixed with 300 μl celite suspension. Followed by centrifuging, the precipitate was washed with 500 μl 40% ice-cold ethanol and washed again with 500 μl ice-cold ddH_2_O, next resuspended with 500 μl ddH_2_O to obtain chitosan solution. The chitosan solution was mixed with 50 μl 10% NaNO_2_ and 50 μl 10% KHSO_4_, incubated at room temperature for 15 min followed by centrifuging. Then, the supernatant was mixed with 10% NaNO_2_ (w/v) and 10% KHSO_4_ (w/v) and centrifuged for 15 min at 4°C. The supernatant was mixed with 20 μl NH_4_SO_3_NH_2_ and vortexed vigorously. Then, 20 μl 3-methyl-2-benzothiazolinone hydrazone hydrochloride hydrate (MBTH) (Sigma-Aldrich, St Louis, MO, United States) was added and heated at 100°C for 5 min. Cooling at room temperature, added 20 μl 0.83% FeCl_3_.6H_2_O solution, and mixed thoroughly. Then, the solution was transferred to a 96-well plate and the absorbance at 650 nm was recorded. The standard curve was described by standard glucosamine solutions (500, 400, 300, 200, 100, 80, 60, 40, 20, and 0 μg/ml). Each epidermis sample was pooled by epidermis from six silkworm prepupae and three epidermis samples were assayed for chitin quantification. This experiment was performed in triplicate.

### Statistical Analysis

Prior to analysis, all the variables were tested for normality with the Kolmogorov–Smirnov test. Data comprising two groups were analyzed using the Student’s *t*-test for unpaired comparisons. Data are shown as mean ± SD. A *P*-value of *<0.05, ^**^0.01, or ^***^0.001 was considered significant, highly or the most highly significant, respectively. All the analyses were conducted by using SPSS software version 19.0 (SPSS Incorporation, Chicago, IL, United States). Figures were drawn using GraphPad Prism version 7 (San Diego, CA, United States) and assembled in Adobe Illustrator CS6.

## Results

### *Exorista japonica* Development in the Host Silkworm

**FIGURE 1:**
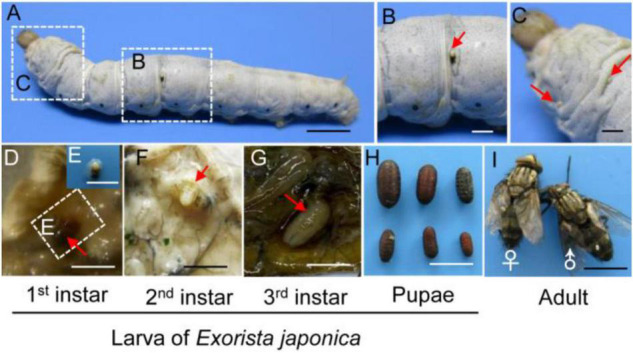
The development stage of *Exorista japonica*. **(A)** *E. japonica* adults laid eggs on silkworm integument at the fifth larval stage. **(B)** A hatched egg indicated by a black-marked respiratory funnel at the point of egg laying (as shown by a red arrow). **(C)** Unhatched eggs without respiratory funnels (indicated by red arrows). **(D,E)** The first instar of *E. japonica* with a melanized sclerotized sheath around the posterior part of silkworm larval body. **(F)** The second instar of *E*. *japonica* attached to the respiratory funnel. **(G)** The third instar of *E*. *japonica* migrated freely in hemocele. **(H)** Pupae of *E*. *japonica* with different sizes. **(I)** Adults of *E*. *japonica*. Bars, 5 mm. Red arrows indicated the tachinid eggs or larva in **(D–G)**.

We initially observed the development of *E. japonica* on silkworms. The mated females laid 2 or 3 heavy-shelled macrotype eggs on cuticle of the fifth-instar silkworm (Figures 1A–C). 38–52% of laid eggs hatched and penetrated the host integument after incubating for approximately 4 days; as soon as they entered, respiratory funnels identified by the presence of black markings due to the melanization of the opening on the cuticle at or near the point of egg laying were formed (Figure 1B and Tables 1, 2). However, 30% of the treated silkworm larvae were not invaded by the hatched tachinid larvae as respiratory funnels could not be observed on host integument (Figure 1C and Table 1). The first and second-instars attached themselves to the respiratory funnel that is a sclerotized sheath derived from host defensive cells, around the posterior part of their larval body, enabling the developing tachinid larvae continuous access to fresh air through a hole in the integument of the host (Figures 1D–F; Valigurová et al., 2014). The third-instar migrated freely within host hemocele (Figure 1G). Around 65% of the hatched larvae could complete their development; these larvae developed visible respiratory funnels that became mature and exited from the host remain, pupated in a day, and the pupa emerged in around 10 days (Figures 1H,I and Tables 1, 2). Interestingly, mature *E*. *japonica* larvae exited from the silkworm pupa and pupated inside the cocoon and the emerged tachinid adults could not exit the cocoon and died inside (Table 3). Multiple larvae can live within the host body and complete larval stages, but the sizes of pupae varied (Figure 1H). This tachinid species completed one generation in less than 20 days when rearing on silkworm larvae (Table 2).

**TABLE 1:** Survival of *Exorista japonica* on the fifth instar *Bombyx mori*.

Number of tachinid eggs/host	Number of hosts treated	% hosts parasitized with one tachinid larva (one funnel)	% hosts parasitized with two tachinid larvae (two funnels)	% tachinid egg hatched	% tachinid puparia emerged^a^	% tachinid pupae emerged^b^
2	50	64.00 ± 5.66	6.00 ± 2.83	38.00 ± 5.66	74.77 ± 5.91	63.31 ± 1.99
3	50	68.00 ± 11.31	18.00 ± 2.83	52.00 ± 2.83	71.34 ± 6.60	65.63 ± 9.01

*^a^Number of pupae/number of hatched larvae.*

*^b^Number of adults/number of pupae.*

**TABLE 2:** Developmental period of *E. japonica* on the fifth instar *B. mori*.

Variable	Value (mean ± SD)
Egg to funnel (days)	4.15 ± 0.37 (*n* = 65)
Egg to pupa (days)	9.13 ± 0.33 (*n* = 55)
Pupa to adult (days)	10.15 ± 0.36 (*n* = 55)
Generation time (days)	19.28 ± 0.45 (*n* = 55)

**TABLE 3:** Number of silkworm cocoon with *E. japonica* pupa inside.

Replicate	Number of hosts parasitized	% cocoon with tachinid pupa inside
Replicate 1	40	40
Replicate 2	35	35
Replicate 3	52	52

### Effects of *Exorista japonica* Parasitoidism on Host Fitness

**FIGURE 2:**
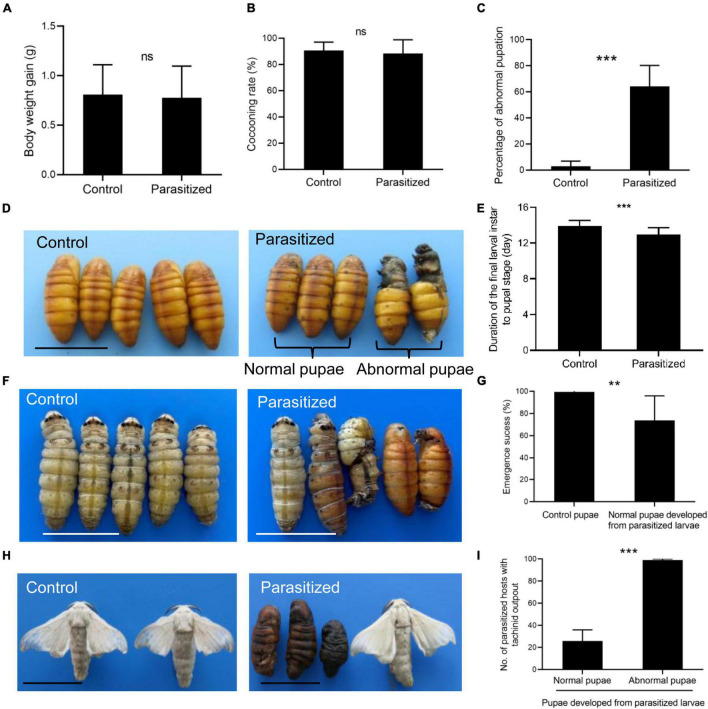
The influences of *E*. *japonica* parasitoidism on silkworm fitness. **(A)** Body weight gain in silkworm larvae between 2 and 4 days after parasitization (DAP). **(B)** The cocooning rate in the control and parasitized host. **(C)** The percentage of abnormal pupation caused by tachinid parasitoidism. **(D)** The morphological changes of parasitized pupae. **(E)** Duration of molting from the fifth larval stage to the pupal stage in nonparasitized and parasitized hosts. **(F)** The developmental stages of nonparasitized and parasitized hosts at 8 DAP. **(G)** The adult emergence rate of control pupae and normal pupae developed from parasitized larvae. **(H)** The morphology of control adults and adults emerged from normal pupae developed from parasitized larvae and consumed pupae. **(I)** The number of pupae that developed from parasitized larvae produced mature tachinid larvae. All the data were presented as mean ± SD with *n* = 3. Significance of the results was determined by the Student’s *t*-test; **P < 0.01, ***P < 0.001, ^ns^not significant. Bars, 2 cm.

We then investigated the effect of *E. japonica* parasitoidism on the host silkworm fitness. The parasitoidism had no effect on host body weight gain and cocooning rate (Figures 2A,B). Interestingly, the parasitized hosts exhibited a significant increase in abnormal pupation rate of 64% and the abnormal pupae showed incomplete removal of the old cuticle of the head (Figures 2C,D, *P* = 0.0003 for Figure 2C). Meanwhile, the larval–pupal transition was significantly advanced (24 h) in the parasitized hosts (Figures 2E,F, *P* = 4.49e−06 for Figure 2E) and a 26% reduction in adult emergence of normal pupae developed from parasitized larvae (Figures 2G,H, *P* = 0.005). Although 36% of the parasitized larvae underwent successful larval–pupal transition and developed into normal pupae, 25% of them could not develop into adults and showed a complete lethality due to tachinid larval feeding and these tachinid larvae completed the larval stage within the host pupae (Figures 2C,I). Therefore, tachinid parasitization-induced growth retardation of host occurred at the prepupal or pupal stage. The larval–pupal molts and pupal–adult transformation were normal in control insects (Figures 2D,G). Expectedly, tachinid larvae were present in all (normal and deformed) the host pupae that did not develop into adults, while absent in host pupae that developed into adults, suggesting that successfully emerged parasitized hosts eliminated tachinid larvae (Figure 2H, *P* < 0.001).

### *Exorista japonica* Parasitoidism-Induced Abnormal Metabolism of Juvenile Hormone and Ecdysteroid

**FIGURE 3:**
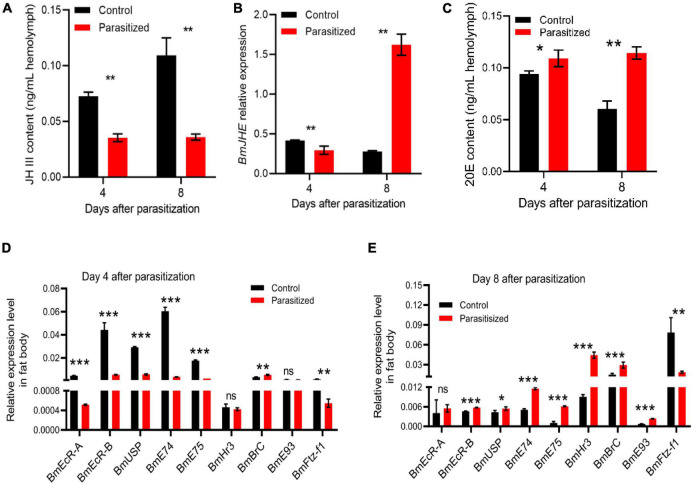
Detection of juvenile hormone (JH) and 20-hydroxyecdysone (20E) titers and the transcription of their responsive genes. **(A)** JH III titers in the hemolymph of silkworm larvae at 4 (silkworms at wandering stage) and 8 (silkworm at prepupal stage) DAP. **(B)** The expression level of *BmJHE* in the fat body relative to *BmActin3*. **(C)** 20E titer in the hemolymph of silkworm larvae at 4 and 8 DAP. **(D)** The expression levels of 20E-responsive genes relative to *BmActin3* in the fat body of silkworms at 4 DAP. **(E)** The expression levels of 20E-responsive genes relative to *BmActin3* in the fat body of silkworms at 8 DAP. All the data were presented as mean ± SD with *n* = 3. Significance of the results was determined by the Student’s *t*-test; *P < 0.05, **P < 0.01, ***P < 0.001, ^ns^not significant.

To investigate the mechanism for host pupation defects caused by *E. japonica* parasitoidism, we, thus, measured the titer of JH and 20E as well as the transcription of their responsive genes in nonparasitized silkworms and parasitized silkworms at 4 (wandering stage) and 8 (prepupal stage) days post-parasitoidism in which the levels of JH and 20E had dramatic changes (Kayukawa et al., 2014). We observed that the titer of JH was dramatically decreased in the hemolymph of parasitized silkworms at 4 (silkworms at wandering stage, also mature larvae) and 8 DAP (silkworms at prepupal stage) (Figure 3A, *P* = 0.001 at 4 DAP, *P* = 0.003 at 8 DAP). Moreover, the transcript level of *BmJHE*, a JH-degrading enzyme, was decreased by 1.5-fold in silkworms at 4 DAP, which was probably achieved by feedback downregulation of *BmJHE* transcript level due to the reduced substrate JH. Meanwhile, *BmJHE* transcription was increased by sixfold in silkworms at 8 DAP, resulting in a dramatic decrease in JH titer in prepupae (Figures 3A,B). Concomitant with reduced JH titer, the titer of 20E was increased in parasitized hosts at both the stages, which stimulated molting (Figure 3C, *P* = 0.039 at 4 DAP, *P* = 0.003 at 8 DAP). Meanwhile, the transcription levels of 20E-responsive genes such as *BmEcR-A*, *BmEcR-B*, *BmUSP*, *BmE74*, *BmE75*, and *BmFtz-f1* were significantly downregulated in the fat body of silkworm larvae at 4 DAP (Figure 3D), while the expression levels of *BmEcR-B*, *BmUSP*, *BmE74*, *BmE75*, *BmHr3*, *BmBrC*, and *BmE93* were elevated in silkworm prepupae at 8 DAP (Figure 3E). Intriguingly, *BmFtz-f1* transcription was dramatically suppressed in silkworm prepupae, suggesting that inhibition of *BmFtz-f1* expression by parasitoidism disrupted host pupariation (Figure 3E).

### *Exorista japonica* Parasitoidism-Induced Silencing of *BmFtz-f1* and Cuticular Protein Genes in Epidermis

**FIGURE 4:**
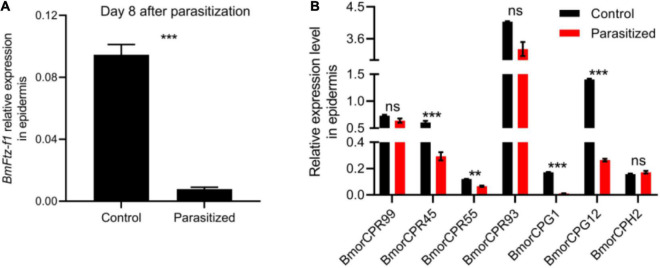
The effect of *E. japonica* parasitoidism on expression of *BmFtz-f1* and cuticular protein genes in host epidermis. **(A)** The expression level of *BmFtz-f1* in host epidermis. **(B)** The expression levels of cuticular protein genes in host epidermis relative to *BmActin3*. All the data were presented as mean ± SD with *n* = 3. Significance of the results was determined by the Student’s *t*-test; **P < 0.01, ***P < 0.001, ^ns^not significant.

Fushi-tarazu factor 1 has been suggested to be a regulator responsible for the stage-specific expression of cuticular protein genes, which contribute to the maintenance and structure of the cuticle during the prepupal stage (Shahin et al., 2016). We then measured the transcriptional levels of *BmFtz-f1* and seven cuticular protein genes (*BmorCPR99*, *BmorCPR45*, *BmorCPR55*, *BmorCPR93*, *BmorCPG1*, *BmorCPG12*, and *BmorCPH2*) that have been determined to be positively regulated by *BmFtz-f1* in epidermis of control and parasitized silkworms at prepupal stage (Suzuki et al., 2002; Shahin et al., 2016). The expression level of *BmFtz-f1* was decreased by 11-fold at 8 DAP (Figure 4A). Meanwhile, the transcriptional levels of *BmorCPR45*, *BmorCPR55*, *BmorCPG1*, and *BmorCPG12* were decreased in epidermis of parasitized hosts, while *BmorCPR99*, *BmorCPR93*, and *BmorCPH2* did not show significant changes (Figure 4B). These results suggested that tachinid parasitoidism led to silencing of *BmFtz-f1* expression, which, in turn, downregulated cuticular protein gene expression, causing malformed cuticles.

### *Exorista japonica* Parasitoidism Caused a Decrease in Trehalose Content and an Increase in Chitin Content

**FIGURE 5:**
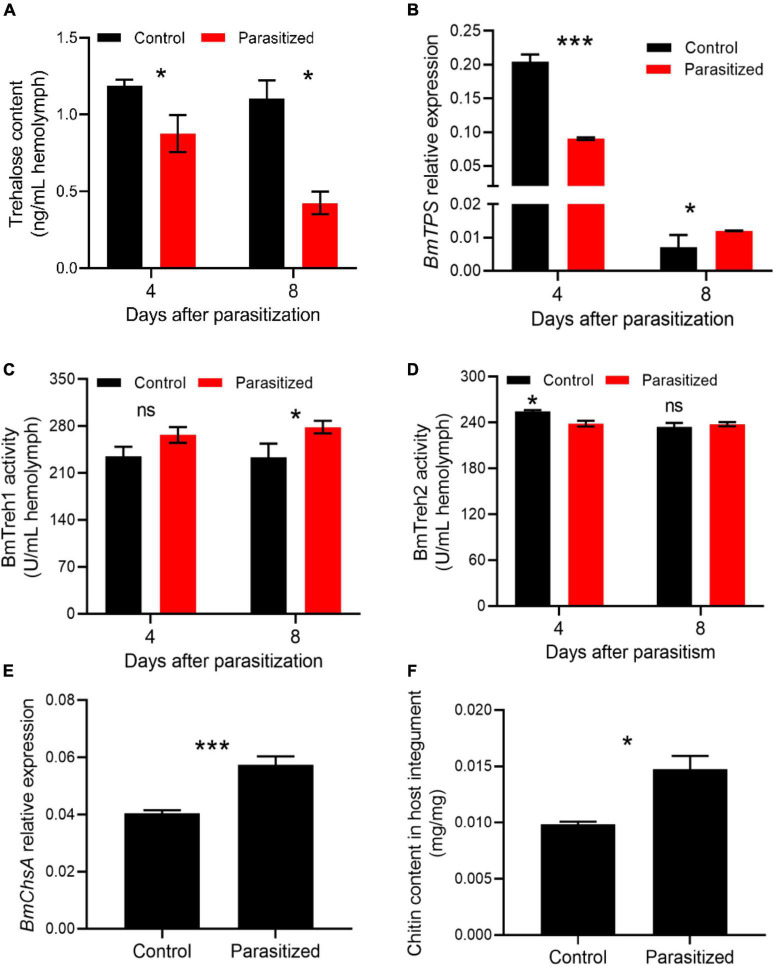
The effect of *E*. *japonica* parasitoidism on trehalose metabolism and chitin synthesis. **(A)** Trehalose content in the hemolymph of silkworm larvae at 4 (silkworm at wandering stage) and 8 (silkworm at prepupal stage) DAP. **(B)** The expression level of *BmTPS* in the fat body relative to *BmActin3*. **(C)** BmTreh1 activity in the hemolymph of silkworm larvae at 4 and 8 DAP. **(D)** BmTreh2 activity in the hemolymph of silkworm larvae at 4 and 8 DAP. **(E)** The expression level of *BmChsA* relative to *BmActin3* in integument of silkworms at 8 DAP. **(F)** Chitin content in integument of parasitized silkworms at prepupal stage. All the data were presented as mean ± SD with *n* = 3. Significance of the results was determined by the Student’s *t*-test; *P < 0.05, ***P < 0.001, ^ns^not significant.

Considering that insect metamorphosis is largely dependent on chitin metabolism (Zhu et al., 2016), we next asked if trehalose metabolism, the starting substrate for chitin biosynthesis, was altered in parasitized hosts. The trehalose content was significantly less in the hemolymph of parasitized insects at 4 and 8 DAP, suggesting a more consumption of trehalose (Figure 5A, *P* = 0.015 at 4 DAP, *P* = 0.042 at 8 DAP). The transcription of trehalose synthesis enzyme *BmTPS* was downregulated in the fat body of mature larvae, but significantly upregulated in prepupae of the parasitized groups, which appeared to result from the feedback induction of trehalose synthesis due to insufficient trehalose resource for pupation (Figure 5B). Moreover, BmTreh1 activity in the parasitized groups was increased, while BmTreh2 activity was comparable to that seen in the hemolymph of control prepupae (Figures 5C,D). We further found that the transcription of Bm*ChsA* was upregulated in the integument of prepupae in the parasitized groups (Figure 5E). Consistent with messenger RNA (mRNA) levels, an increased content of chitin in prepupal integument was observed (Figure 5F, *P* = 0.028).

## Discussion

Tachinids role as enemies of insect herbivores and in the structure and dynamics of natural ecosystems have been largely unexplored. Several large-scale caterpillar-rearing programs focusing on exophytic macrolepidoptera have, however, shown that parasitoidism by tachinids is often equivalent to and sometimes greater than that by hymenopteran parasitoids in the field (Elkinton and Boettner, 2012; Weber et al., 2021). Thus, the tachinid has a great potential in biological control against different pests. However, *in vitro* rearing of tachinids with artificial diets has been proven to be difficult because of their complex interactions with the host and specific nutritional needs (Dindo and Grenier, 2014). Currently, tachinids for biocontrol are mainly reared on their hosts, thereby producing quality organisms with a suitable host at a reasonable cost is necessary. In this study, we found that yields of *E. japonica* rearing on *B. mori* were comparable with on *P. separata* or *Galleria mellonella*, suggesting that *B. mori* could be a potential host for mass production of this tachinid parasitoid (Dindo and Nakamura, 2018; Zhao et al., 2020). Moreover, the fly larvae pupated and emerged inside the cocoon, the emerged adults could not exit from the cocoon, and, thus, rearing on the silkworm can prevent the parasitoid from escaping and threatening silkworm rearing. Additionally, understanding the occurrence regularity of tachinid parasitoids and the effects of parasitization on silkworms would provide guidance to the sericulturists for the optimal selection of the time for rearing silkworms such as avoiding rearing during tachinid outbreak season.

The evolution of insect host-parasitoid interactions involves development of intimate physiological and ecological interactions. Owing to resource competition among the antagonists, both the host and parasitoid can influence the growth and development of each other. In some interactions, the host may dominate, while in others, the parasite may dominate. A few tachinid species can induce a precocious wandering or prepupal stage in host larvae as it has been reported in *Eucelatoria bryani* and *C. dorsalis* (Reitz and Nettles, 1994; Walker, 2011). This usually results from a decrease in JH titer and an increase in JHE activity and an altered ecdysteroid titer. In addition, the most common effect of hymenopteran parasitoids in their hosts was induction of developmental arrest in the last stadium before emergence of the parasitoids, resulting from a hormone imbalance (i.e., ecdysteroids and JH) (Soller and Lanzrein, 1996; Pfister-Wilhelm and Lanzrein, 2009; Jones et al., 2010; Asgari and Rivers, 2011). In this study, we showed that the host parasitized by the tachinid *E. japonica* could develop normally up to the pupal stage, but then became arrested at the pupal cell formation stage, which led to incomplete pupation. Interestingly, we observed a reduction in JH titer, an increase in 20E titer, and inhibited *BmFtz-f1* expression in parasitized hosts, suggesting that host steroid hormone signaling was regulated by the tachinid parasitoid. The duration of immature (larvae or pupae) insect development time was frequently shortened by abiotic stresses such as heat stress or pesticide exposure, which probably could be viewed as the attempt of the insect to grow fast to complete reproduction (Guo et al., 2013; Cui et al., 2017). We, therefore, deduced that the host and parasitoid might compete for resources for their own growth, namely, the host attempted to complete development earlier and, thus, needed more resources to activate 20E synthesis, while the parasitoid inhibited 20E signaling in the host through silencing *BmFtz-f1* expression and further blocked host resource investment on pupation. Also, it is possible that the tachinid parasitoid targets host endocrine system through synchronizing its development with the development of the host to complete larval development. Insects that are attacked by parasitoids, either hymenopteran or tachinid, possess an immune system that can eliminate invading foreign organisms. One cellular immune reaction against invading parasitoid eggs and larvae is encapsulation with hemocytes followed by melanization (Yamashita et al., 2019; Yang et al., 2021). Recent studies identified enhanced expression of innate immunity components of toll and melanization pathways, cytokines or proteolytic enzymes, and oxidative reactions in *B. mori* following *Exorista sorbillans* parasitoidism (Pradeep et al., 2013; Anitha et al., 2014; Jayaram et al., 2014; Makwana et al., 2017; Pooja et al., 2017; Xu et al., 2019). We showed here that around 35% parasitized larvae have undergone successful larval–pupal transition and around 25% of the successful pupated parasitized hosts could eliminate tachinid larvae either in larval or pupal stage and undergo successful eclosion. Unlike female parasitoid wasps that deposit their eggs into the host and inject immune-suppressive venoms to allow their offspring to develop inside the host hemocele, the tachinid larvae may escape host encapsulation by building respiratory funnels, hiding in a host muscle or ganglion, or secreting factors or effectors against host immunity in the absence of materials from their parents (Hayakawa, 1986; Kim-Jo et al., 2019; Yang et al., 2021). Thus, the “arms race” between tachinid parasitoid and host leads to coevolution of antiparasitic defense and parasitic mechanism, which are warranted to be investigated.

Similar to the disabilities caused by tachinid parasitoidism, phenotypic defects such as arrested development and abnormal pupation have been reported in insects whose ecdysteroid synthesis was dysregulated or whose ecdysteroid-mediated signaling was inhibited following pesticide application (Chen et al., 2020; Zhang et al., 2021). Generally, increased 20E level is supposed to normally induces the 20E-responsive genes. In this study, though 20E titer increased at 4 days postparasitization, the expression of most 20E-responsive genes was decreased, suggesting that the expression of 20E-responsive genes probably was inhibited by parasitoid factors. At 8 DAP, consistent with increased 20E titer, tachinid parasitoidism led to a significant increase in 20E titer and subsequent elevated transcription of the 20E-responsive genes in hosts, while *BmFtz-f1* expression showed a decrease. Ftz-f1 is a unique transcription factor, which is induced by a 20E pulse; the high level of Ftz-f1 expression coincided with the high level of 20E titer in insects during each molting and larval–pupal and pupal–adult stages (Fahrbach et al., 2012). The defective phenotypes caused by *Ftz-f1* downregulation have been reported in many insects such as *Drosophila* spp. and *Blattella germanica* (Cruz et al., 2010; Sultan et al., 2014). Clearly, in *Drosophila*, the early nuclear receptor E75B works as an Ftz-f1 corepressor through a HR3-E75B interaction, whereas HR3 positively regulates Ftz-f1 expression through direct interaction during the prepupal stage, which, thus, potentiates 20E action that regulates the normal fly development (Kageyama et al., 2010). In parasitized *B. mori*, the transcript levels of *BmE75B* and *BmHR3* were all upregulated; we, therefore, speculate that the putatively increased BmE75B protein and BmFtz-f1 probably compete for binding to BmHR3 and BmE75B/BmHR3 heterodimers are preferentially formed over BmFtz-f1/BmHR3, which, in turn, inhibits a positive regulation of BmFtz-f1 by BmHR3, thereby resulting in the production of defective pupae.

Numerous cuticular protein genes are regulated by ecdysone-responsive transcription factors such as Ftz-f1 and have been proven to be associated with larval–pupal transformation in insects (Shahin et al., 2022). Metamorphosis is adversely affected by reduced mRNA expression of cuticular protein genes, which has been studied in many insects, such as *B*. *mori*, *Tribolium castaneum*, and *Locusta migratoria* (Mun et al., 2015; Shahin et al., 2016; Zhao et al., 2019). Ftz-f1 can bind to the upstream region of these cuticular protein genes and increased their promoter activity, stimulating the formation of new cuticular layers during larval–pupal ecdysis (Shahin et al., 2022). In *B. mori*, expression of cuticular protein genes such as *BmorCPG1* and *BmorCPR99* is induced by an ecdysone pulse through *BmFtz-f1* (Suzuki et al., 2002; Shahin et al., 2016). In this study, the expression of *BmFtz-f1* in silkworm epidermis was significantly reduced after parasitization. Accordingly, the expression of *BmFtz-f1*-dependent cuticular protein genes was also inhibited. Therefore, tachinid parasitization-mediated knockdown of *BmFtz-f1* expression led to silencing of cuticular protein genes in the host epidermis, resulting in cuticle defects during prepupal–pupal transition.

Trehalose, the major blood sugar synthesized by TPS, is considered as the initial substrate for chitin synthesis (Tang et al., 2018). In this study, tachinid parasitoidism caused decreased trehalose content in host hemolymph, which mainly resulted from larval feeding of host fat body and hemolymph that produced and stored trehalose, respectively. Meanwhile, we deduced that the parasitized host required sufficient trehalose as substrates for chitin synthesis to support pupation and, thus, a feedback induction of *BmTPS* transcription occurred. However, host demands for trehalose could not be compensated. The downstream chitin synthesis was increased in prepupal integument probably due to the maximum utilization of trehalose in parasitized hosts, which showed enhanced trehalase activity. In particular, during host prepupal–pupal transition, the tachinid larva, readily to develop to the third stage, has extensively exploited host resources, leading to less nutrient resource supply for host pupation.

## Conclusion

In conclusion, we demonstrate that tachinid parasitoidism causes reduced developmental duration by increasing 20E levels and lowering JH titers. Meanwhile, by silencing the expression of host *BmFtz-f1* and cuticle protein-coding genes, *E. japonica* prevents the normal molting cycle. These findings suggest the target mechanism of tachinid parasitoids and provide new clues to host-/tachinid-parasitoid relationships as well as to application of tachinid parasitoids in biological control.

## Data Availability Statement

The original contributions presented in the study are included in the article/Supplementary Material, further inquiries can be directed to the corresponding authors.

## Author Contributions

BL, JW, and M-LD conceived and designed the experiments, analyzed the data, and wrote the manuscript. M-LD, W-TY, X-JJ, and PF investigated the phenotypic changes. M-LD, W-TY, and Q-YZ did gene expression analysis and measurement of hormonal, trehalose, and chitin metabolism. H-NS and F-CL provided technical assistance. All authors contributed critically to the drafts and gave final approval for publication of the manuscript.

## Conflict of Interest

The authors declare that the research was conducted in the absence of any commercial or financial relationships that could be construed as a potential conflict of interest.

## Publisher’s Note

All claims expressed in this article are solely those of the authors and do not necessarily represent those of their affiliated organizations, or those of the publisher, the editors and the reviewers. Any product that may be evaluated in this article, or claim that may be made by its manufacturer, is not guaranteed or endorsed by the publisher.

## Footnotes

1
https://www.cabi.org/isc/datasheet/9514
