# Supplementary material for: Effect of Tachinid Parasitoid Exorista japonica on the Larval Development and Pupation of the Host Silkworm Bombyx mori
Source: Front Physiol. 2022 Feb 16;13:824203. doi: 10.3389/fphys.2022.824203 (PMC8889078; doi:10.3389/fphys.2022.824203)
Supplement: Supplementary file 1 [file Presentation_1.pdf]

Supplementary Figure 1. Experimental design for silkworm tissue collection. L5D1: 1-day-old 5th instar, L5D7: 7-day-old 5th instar, L5D8: 8-day-old 5th instar, RF: respiratory funnel, DAP: day after parasitization, W: wandering stage, PP: prepupal stage, P: pupal stage.

Supplementary Figure 1

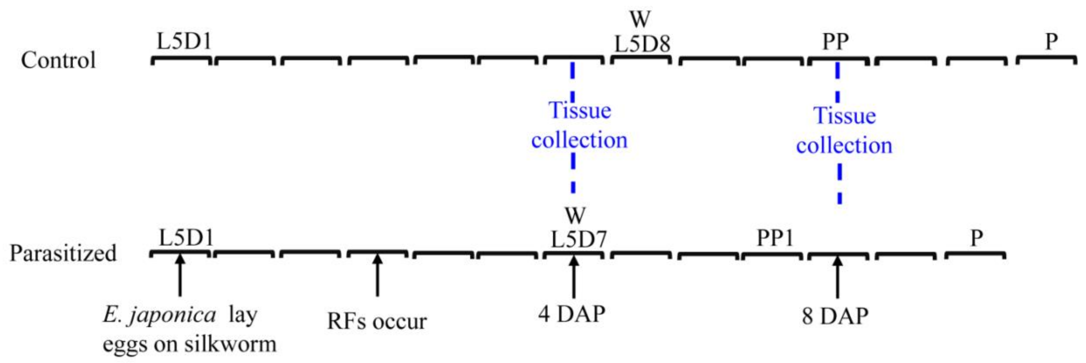

Supplementary Table 1 Primers used in this study for RT-qPCR analyses.

Gene name	NCBI Gene ID/GenBank no.	Primer sequences (5′ to 3′)	Length of product (bp)	Amplification efficiency (%)
BmActin3	100145915	F: CGGCTACTCGTTCACTACC<br>R: CCGTCGGGAAGTTCGTAAG	147	99.87
BmEcR-A	692756	F: CTATCCTCAGGCAGCTACGG<br>R: GTGCAGGACCTTTCTTCTGC	152	104.13
BmJHE	AY489292.1	F: TTCGCGTTTGGATCTGGTGA<br>R: GACCATTGTTTCCGGGGACT	145	98.86
BmEcR-B	692756	F: AGACGCCGCTGGTCTAATAA<br>R: ATTCCGTCATCGTAGCTCCA	169	95.74
BmUSP	693034	F: AATGTCGGTAACTGCGTTGA<br>R: TCGAGTTCAACATTGGGTGT	144	98.99
BmE74	693011	F: TACCTGTGGGAGTTCCTGCT<br>R: TGTCTGGCTTGTTCTTGTGC	151	106.03
BmE75	692595	F: CCCAAGAATGACAAATTCACG<br>R: TGCCCATTGAGACAGATGAT	114	92.45
BmHr3	692562	F: ACCGAGACTGAACTGGCTCT<br>R: GGTATCTTGGCCAGGAGTGT	191	98.79
BmBrC	692523	F: AAGACGTGGCGTACACAGAC<br>R: TCAGGAATGAGGACAAGCTG	93	99.35
BmE93	101737038	F: CCTTCGTCTCCAAGTCCTGT<br>R: TGCTATTCGATGCTCCATCT	142	97.98
BmFtz-f1	693070	F: ATGGTCTGTTATGCTGGTT<br>R: ATTGAAGTGGTCGGCTAAT	145	102.09
BmTPS	101741435	F: TCGGTATCGAAGGCATCACG<br>R: CAACGACCTTGTCTTGCAGC	111	99.04
BmChsA	100884166	F: ACAAGAGGCTCGCATAGCAG<br>R: CCAGACCACGTGAAGCTGAT	136	93.72
BmorCPG1	BR000422	F: GATACGGCGGCTCTGTCAT<br>R: GGGCTGGGACACTACTTTGT	125	98.43
BmorCPR45	BR000546	F: ATCAGGAACAGGTCATCAATCC<br>R: TTATGTAGCCCTGCTCTTCAGC	90	95.28
BmorCPR55	BR000556	F: ATGGGTATATCGACGAGAGTGG<br>R: CGTTGGAAGACGGTTCGTG	129	108.66
BmorCPR99	BR000600	F: AAGCTCGCTGTTGCTACCC<br>R: TAGCGGAGTGGAGAAGTGTG	134	99.14
BmorCPR93	BR000594	F: GTGAATACTCCCTCCTGCAACC<br>R: AATATCACAGATCGAGCAGACTTC	148	94.86
BmorCPG12	BR000433	F: CAGCATCATGAGACCAATGC	114	96.42

		R: GGTTCCTTATCGGTCACTGC		
BmorCPH2	BR000452	F: CCGAAGTGCACGATGAGAG	106	101.23
		R: CGAGTCCGTGGCTGTAGTG		
